# Seizure Susceptibility Corrupts Inferior Colliculus Acoustic Integration

**DOI:** 10.3389/fnsys.2019.00063

**Published:** 2019-11-06

**Authors:** Hyorrana Priscila Pereira Pinto, Eric Levi de Oliveira Lucas, Vinícius Rezende Carvalho, Flávio Afonso Gonçalves Mourão, Leonardo de Oliveira Guarnieri, Eduardo Mazoni Andrade Marçal Mendes, Daniel de Castro Medeiros, Márcio Flávio Dutra Moraes

**Affiliations:** ^1^Núcleo de Neurociências, Departamento de Fisiologia e Biofísica, Instituto de Ciências Biológicas, Universidade Federal de Minas Gerais, Belo Horizonte, Brazil; ^2^Centro de Tecnologia e Pesquisa em Magneto Ressonância, Programa de Pós-Graduação em Engenharia Elétrica, Universidade Federal de Minas Gerais, Belo Horizonte, Brazil

**Keywords:** excitatory-inhibitory imbalance, wistar audiogenic rat, cognitive functions, steady-state evoked response, transient evoked response

## Abstract

Evidence suggests that the pathophysiology associated with epileptic susceptibility may disturb the functional connectivity of neural circuits and compromise the brain functions, even when seizures are absent. Although memory impairment is a common comorbidity found in patients with epilepsy, it is still unclear whether more caudal structures may play a role in cognitive deficits, particularly in those cases where there is no evidence of hippocampal sclerosis. This work used a genetically selected rat strain for seizure susceptibility (Wistar audiogenic rat, WAR) and distinct behavioral (motor and memory-related tasks) and electrophysiological (inferior colliculus, IC) approaches to access acoustic primary integrative network properties. The IC neural assemblies’ response was evaluated by auditory transient (focusing on bottom-up processing) and steady-state evoked response (ASSR, centering on feedforward and feedback forces over neural circuitry). The results show that WAR displayed no disturbance in motor performance or hippocampus-dependent memory tasks. Nonetheless, WAR animals exhibited significative impairment for auditory fear conditioning (AFC) along with no indicative of IC plastic changes between the pre-conditioning and test phases (ASSR coherence analysis). Furthermore, WAR’s IC response to transient stimuli presented shorter latency and higher amplitude compared with Wistar; and the ASSR analysis showed similar results for WAR and Wistar animals under subthreshold dose of pentylenetetrazol (pro-convulsive drug) for seizure-induction. Our work demonstrated alterations at WAR IC neural network processing, which may explain the associated disturbance on AFC memory.

## Introduction

Cognitive functions are dependent on the integration of multiple and distributed neural populations across brain structures (Ward, 2003), but how exactly information is transferred between different processing networks from region to region is still a matter under study. The rhythmic activity of neuronal ensembles creates oscillations, clearly discernible in local field potential (LFP) recordings, that not only maintain a sustained pattern of discharge throughout a period of time but may also produce a time-multiplexing framework, constituting temporal windows of opportunity for recruiting facilitated micro-domain circuits. In fact, phase synchronization and the phase-amplitude correlograms of distinct oscillatory circuits between different structures have been proposed as a functional connection marker among neural networks and a key to access ensembles communication (Varela et al., 2001; Tort et al., 2009). Several metrics of similarity have been developed in order to provide parameters to quantify the dynamics of the functional connectome (Bastos and Schoffelen, 2015), thus identifying both anatomically and temporally the transient exchange of information required for proper sensory-motor integration. Such a framework may provide a very efficient way (i.e., minimizing the number of connections) for transferring information throughout anatomically sequenced pathways of information processing (Buzsáki, 2006), but could also lead to nonspecific network recruitment if lacking proper homeostatic control. Of interest here, the pathophysiology associated with epileptic susceptibility may also disrupt the proper propagation of such specific discharge patterns leading to dysfunctional neural circuitry and memory impairment.

Epilepsy is a common brain pathology that affects approximately 1% of the world population, characterized by the occurrence of recurrent seizure episodes (Fisher et al., 2005). Classically considered as an excitatory-inhibitory imbalance disease, epilepsy is associated with the disturbance of network synchronization that ultimately leads to the ictal hypersynchronous state (Medeiros and Moraes, 2014). The inhibitory synapses play a paramount role in brain rhythm generation by pacing the oscillating activity and supporting local and large-scale network synchrony (Roux and Buzsáki, 2015). Thus, it would be expected that Epilepsy, even outside of the ictal state, would most likely compromise the functional synchronization of neural circuitry; thus, leading to neurological disturbances and associated cognitive comorbidities (Uhlhaas and Singer, 2006). Several studies have reported that epileptic patients often suffer from weakened cognitive functions, such as memory impairment, that negatively impact the quality of life (Perrine et al., 1995; Giovagnoli et al., 2014). Nevertheless, it is still not clear whether more caudal structures may play a role in the network integration corruption that leads to the cognitive deficits, particularly in those cases where there is no evidence of hippocampal sclerosis (e.g., Temporal Lobe Epilepsy). It is important to highlight that the aforementioned System’s View of Epilepsy (Bertram, 2013; Bui et al., 2015), based on the dysfunctional connectome, tackles the problem from an unorthodox point of view that distances itself from the strongly grounded idea of foci, neuron or even molecular origin for the disease (Devinsky et al., 2018).

In order to test the impact of a subclinical (i.e., in terms of seizure induction) inhibitory system disturbance on network synchronicity and cognitive functions, we used a genetically selected strain of animals (Wistar audiogenic rat, WAR) that develops reflex seizures provoked by high-intensity acoustic stimulation (110 dB; Garcia-Cairasco et al., 1992). Additionally, WAR animals display low threshold to pharmacological (pilocarpine and pentylenetetrazol—PTZ) and electrically induced seizures (Scarlatelli-Lima et al., 2003), indicating an inherent imbalance of excitatory-inhibitory tonus making them seizure-prone rats. However, it is important to highlight that WARs present no recurrent and spontaneous seizures (Castro et al., 2017), quite suitable for our study. Therefore, the objective of this work was to evaluate WAR’s performance at memory-related tasks and the information processing in the primary acoustic pathway (inferior colliculus, IC), regarding both bottom-up and top-down modulation changes. The study employed transient (i.e., focusing on bottom-up processing) and the auditory steady-state response (ASSR), an electrophysiological recording methodology that investigates neuronal assemblies integration (Picton et al., 2003; Rabelo and Schochat, 2011). The ASSR evaluates the synchronous neural activity at specific frequencies based on the amplitude modulation of a sound stimulus (Brenner et al., 2009; Rabelo and Schochat, 2011) in short, it provides a specific spectral signature to structures processing the amplitude-modulated sound stimuli. In addition, the real-time ASSR neural entrainment may even assess the continuous feedforward and feedback forces over circuitry processing (Lockmann et al., 2017); thus, allowing the evaluation of physiological and abnormal network coupling (O’Donnell et al., 2013) and signal propagation throughout the sensory processing neural circuitry.

The results show that WAR’s IC response to amplitude-modulated sound stimuli is similar to Wistar animals under the subthreshold dose of pentylenetetrazol (pro-convulsive drug) for seizure-induction. And, despite no disturbance for hippocampus-dependent memories (object recognition and contextual fear conditioning), WAR animals displayed significant impairment for auditory fear conditioning (AFC) along with no indication of IC plastic changes between the pre-conditioning and test phases (ASSR coherence analysis). Our work demonstrated that WAR’s seizure prone neural network displays alterations at the functional connectome level of the primary acoustic pathway; which may explain the associated auditory-related memory impairment observed for cued conditioning experiments.

## Materials and Methods

### Subjects

Male Wistar rats (weighing 300–320 g) were supplied by the Biotério do Instituto de Ciências Biológicas 2 (BICBIO 2) vivarium and WARs by our own breed. Both strains were housed under controlled environmental conditions (22 ± 2°C), with a 12:12-h light-dark cycle and free access to food and water. WAR animals were previously screened by three acoustic stimuli (48-h interval) and all animals at this study presented at least one episode of the tonic-clonic seizure (0.85 severity index; Garcia-Cairasco et al., 1992). The same experimenter performed all the behavioral tasks in order to decrease the animals’ stress for novelty conditions. The experiments have been approved by the Ethical Committee for the Use of Animals (CEUA)—Universidade Federal de Minas Gerais—under license number 112/2014. CEUA directives comply with the National Institutes of Health (NIH) guidelines for the care and use of animals in research. It is essential to highlight that the “motor assessment” investigations evaluated the same Wistar and WAR animals, starting with the open field test and followed by the rotarod paradigm. Each other experiment employed a different set of animals.

### Motor Assessment

#### Rotarod Test

The rotarod test required animals to balance on an elevated constant-speed rotating rod. The endpoint measures were the latency for the first fall and the total number of falls (180 s trial). Rats (Wistar *N* = 10, WAR *N* = 7) were initially trained in the apparatus at a 5-rpm speed (training session) and, 24 h later, tested by three trials (30 min interval) in a 25-rpm velocity (test session). For statistical purposes, the average of the test session trials was used for group comparisons.

#### Open Field Test

Rats (Wistar *N* = 10, WAR *N* = 7) were placed at the center of an unfamiliar acrylic box (60 × 60 × 40 cm) located in an isolated room (similar light and temperature conditions of the housing room) to freely explore the apparatus. The behavioral test was video recorded, and the distance traveled measured by the Any-maze^®^ software for 5 min.

### Hippocampus-Dependent Memory Assessment

#### Novel Object Recognition

**Figure 1:**
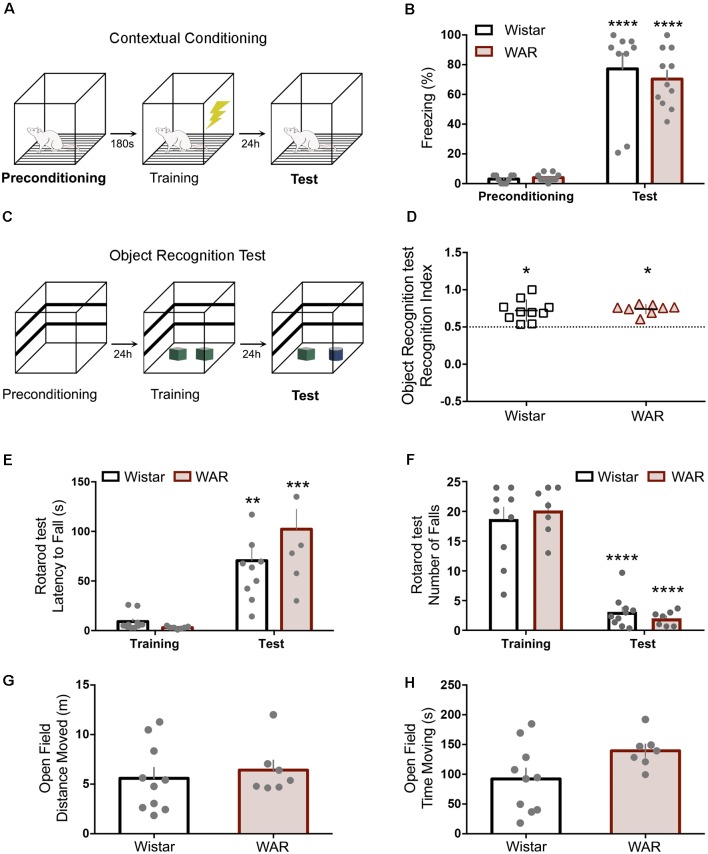
Wistar audiogenic rat (WAR) animals present no motor or hippocampus-dependent memory deficits. **(A,B)** Contextual conditioning task schematic and preconditioning-test freezing behavior percentage. **(C,D)** Object recognition test protocol and recognition index statistically superior chance (50%) for both strains *p < 0.05. **(E,F)** Latency for the first fall and the number of falls on rotarod training and test. **(G,H)** Open field test presented no difference between strains. **p < 0.01, ***p < 0.001 and ****p < 0.001 for session comparisons.

The paradigm aimed to evaluate the rat’s natural interest in exploring not-familiar objects (Figure 1B). Animals (Wistar *N* = 10, WAR *N* = 8) were initially habituated to a rectangular acrylic box (50 × 40 × 20) for 10 min. Twenty-four hours later, rats were re-habituated for 1 min and then allowed to freely investigate (10 min) two identical objects placed inside the apparatus. The next day (test phase), the same protocol was repeated, but one object replaced by a not-familiar item. The recognition index was calculated by the time exploring the not-familiar object (sniffing or nose-touching) over the total time of exploration.

#### Contextual Fear Conditioning

Animals (Wistar *N* = 9, WAR *N* = 11) were placed in a transparent acrylic box with a floor containing stainless-steel grid rods (0.5 cm in diameter, 1 cm separation) for 180 s exploration (Figure 1A). In sequence, rats received three foot-shocks (unconditioned stimulus, US: 0.4 mA for 2 s) with intervals of 120 s (training phase), remaining inside the box 1 min after the last US. Twenty-four hours later, rats returned to the same box for 120 s, but no foot-shock (test phase). Both periods were recorded to offline quantification of the freezing behavior (absence of movements, except the respiratory; Mourão et al., 2016).

### Stereotaxic Surgery

All animals from the protocols “IC acoustic integration” and “IC sound processing” were submitted to the IC electrode implantation for LFP recordings. Rats were initially anesthetized with ketamine (80 mg/kg) and xylazine (10 mg/kg) administered intraperitoneally and positioned in a stereotaxic frame (Stoelting Co., Wood Dale, IL, USA). The scalp was injected with subcutaneous anesthesia (lidocaine, 5 mg/kg) before incision. Monopolar electrodes, made of stainless-steel Teflon-coated wires (Model 791600, A-M Systems Inc., Carlsborg, WA, USA), were implanted in the right IC (AP: −9.0 mm referenced from the Bregma, ML: −1.4 mm, DV: −4.0 mm). In addition, surface micro-screws were positioned over both frontal lobes for reference and ground. The electrodes and micro-screws were then soldered to a connector (RJ11–6 pins), which in turn was fixed to the rat’s skull with dental acrylic cement.

### IC Acoustic Integration

The IC integration processing was performed by LFP recording during the classical AFC task on Wistar (Paired *N* = 6, Unpaired *N* = 5) and WAR (Paired *N* = 7, Unpaired *N* = 6) animals.

#### Auditory Fear Conditioning (AFC)

**Figure 2:**
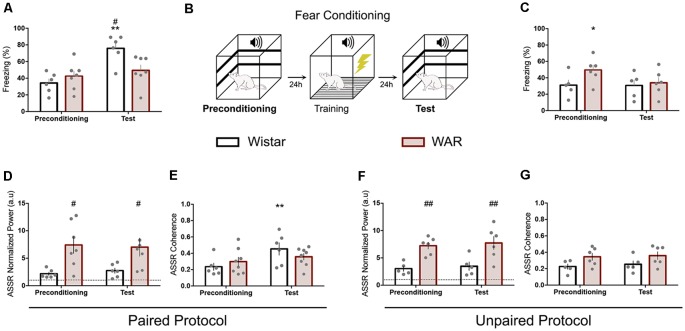
WAR animals presented similar behavioral and electrographic responses at the Auditory fear conditioning (AFC) phases. Fear conditioning task sequence **(B)** and freezing quantification for paired **(A)** and unpaired **(C)** protocol. Paired **(D–F)** and Unpaired **(E–G)** auditory ASSR power and coherence. The dashed lines at **(D,F)** represent the baseline value. *p < 0.05 and **p < 0.01 for session comparisons and ^#^*p* < 0.05 and ^##^*p* < 0.01 for group statistical significance.

The fear conditioning protocol was divided into three consecutive phases (24 h interval): preconditioning, training, and test (Figure 2B). At all stages, the conditioned stimulus (CS) was set as a 10-kHz pure tone modulated in amplitude (100% modulation depth) by a sine wave of 53.7 Hz. The CS intensity was set to 85 dB SPL, measured at the center of the conditioning chamber (Brüel & Kjær type 2238 sound level meter). The CS was randomly presented (interval between 30 and 180 s) five times for 30 s. The preconditioning and test phase were performed in a black acrylic box (30 × 30 × 30 cm) located in an isolated room. During the training phase, animals were placed in a transparent acrylic box (separate room) and received five foot-shocks (0.4 mA for 2 s) as the US. The US was applied at the last 2 s of CS for the paired groups or during the silent periods (20 s minimum separation from the sound stimulus) for the unpaired groups. The preconditioning and test phases were video-recorded for offline quantification of freezing, defined as no movements, except breathing, for a minimum of 3 s within each 5 s time epoch (six epochs for the 30 s; Mourão et al., 2016).

#### LFP Recording

The IC LFP signal was amplified (10k gain) and filtered (1–2,000 bandpass filter) by a signal conditioner. A different channel recorded a sine wave of 53.7 Hz (same filters but no amplification) to indicate the sound stimulation timestamp. Data were sampled at 12 kHz for all channels by using Tucker-Davis Technologies (PZ2) system.

#### LFP Analysis

The preconditioning and test LFP recording were analyzed employing power and coherence within the acoustic modulation frequency (53.71 ± 0.6 Hz). The signal power was calculated by the standard MATLAB function *spectrogram* (short-time Fourier transform-STFT; 32768 points hamming window) along each sound stimulation period and normalized by the respective baseline value (the 30 s before the acoustic stimulus). The LFP coherence between channels was evaluated by using the algorithm *mscohere* (1.34-s windows and 50% overlap) from MATLAB’s Signal Processing Toolbox.

### IC Sound Processing

#### Experimental Groups

Groups were formed according to the strain and the drugs used. Wistar (Total *N* = 11) and WAR (Total *N* = 10) animals received intraperitoneal (I.P) injections 30 min before the LFP recording (48 h interval) of saline (0, 2 ml; Saline-Wistar and Saline-WAR groups) and diazepam (DZP-4 mg/kg—DZP-Wistar and DZP-WAR groups). The Wistar rats were also submitted to pentylenetetrazol (PTZ) injection (40 mg/kg—PTZ-Wistar group). The pharmacological manipulation aimed to modulate the neural excitatory tonus on both strains, toward a higher excitability level by the use of PTZ [gamma-aminobutyric acid type A inhibition (Squires et al., 1984)] or a greater inhibitory state by diazepam (Calcaterra and Barrow, 2014). The rationale for pharmacological intervention was to use drugs with opposing-effects on neural network excitability, that is: DZP used to reverse WAR intrinsic hyperexcitability and PTZ to enhance Wistar excitability. It is essential to highlight that the PTZ dose employed in this study was subthreshold for seizure-inducing (Reeta et al., 2011; for PTZ-Wistar group).

#### Acoustic Stimulation

**Figure 3:**
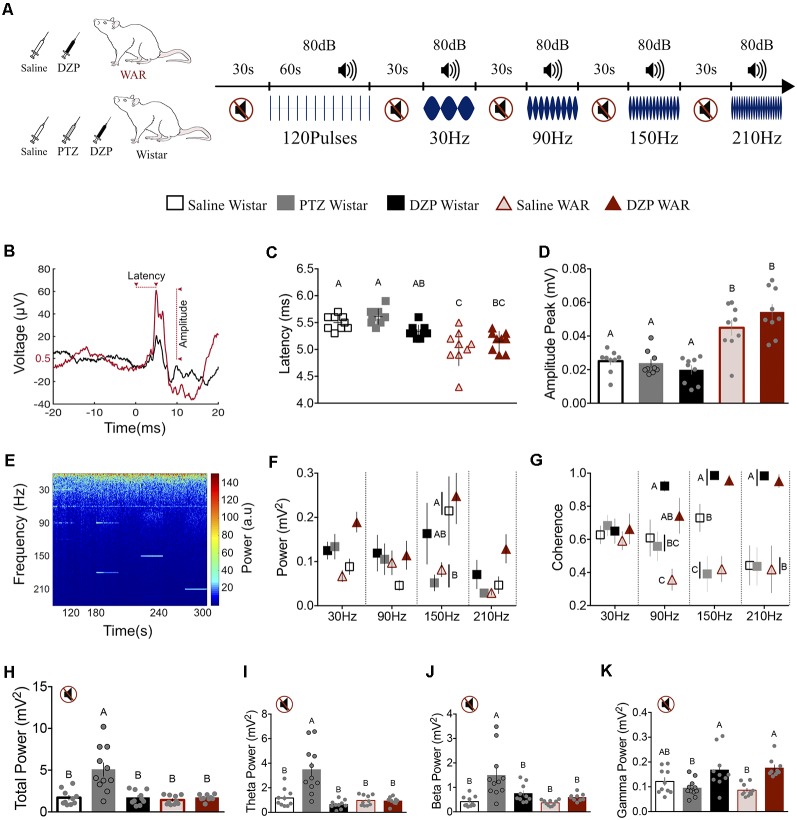
Neuronal excitability level and rat strain modify the inferior colliculus (IC) basal activity **(H–L)** and the sound-evoked responses by transient **(B–D)** and ASSR **(E–G)**. **(A)** Illustrative protocol sequence. **(B)** Representative transient evoked response (120 averaged pulses) from Saline Wistar (black) and Saline WAR (red) group. The arrows indicate the start-end point for WAR Saline latency and amplitude. The Y-axes 0.5 μV refers to the WAR Saline baseline value determined by averaging the 20 ms-time-window prior to sound presentation. Peak amplitude measurement was determined against this baseline. **(C,D)** Transient response peak latency and amplitude. **(E)** IC ASSR demonstrative spectrogram. **(F,G)** IC power and coherence according to the sound stimulation modulation frequencies (30–210 Hz). **(H–K)** IC basal activity during the silent period; Total Power and Theta, Beta and Gamma frequency bands. Different letters represent statistical significance *p* < 0.05.

The IC activity was evoked by transient and steady-state sound stimuli (80 dB SPL measured at the center of the recording chamber—Brüel & Kjær type 2238 sound level meter—Figure 3A). The transient stimulus consisted of 120 tone bursts of 10 kHz (40 ms duration: 1 ms rise and down-45° inclination and 38 ms plateau) for a period of the 60 s (two clicks per second-460 ms interpulse interval). The steady-state sound was a 10 kHz pure tone modulated in amplitude (100% modulation depth) by the following frequencies: 30, 90, 150 and 210 Hz (30 s duration for each). Periods of the 30 s of silence (Figure 3A) spaced the transient stimuli and each modulation frequency. The sound stimulation was designed by using Adobe Audition 5.0, digitally recorded (44.1-kHz sampling rate, 16-bit resolution) and saved in one channel of a stereo WAV file format. The other channel carried a reference soundtrack used to synchronize the IC activity and the acoustic stimulation, consisting of a square wave for each transient pulse and sine wave at the steady-state modulatory frequencies (30, 90, 150 and 210 Hz).

#### LFP Recording

The IC LFP was amplified (10k gain) and filtered (from 1 to 2 kHz bandpass filter) by a signal conditioner (Cyberamp 380, Axon Instruments). The reference soundtrack was also recorded on a different channel (same filters but no amplification) to obtain the exact time of the sound stimuli. Data were sampled at 10 kHz for both channels using an A/D converter (National Instruments Inc., mod NIDAQ 6023E) and stored on a computer hard disk for offline analyses.

#### LFP Analysis

LFP recordings from awake and seizure-free animals were analyzed by MATLAB^®^ 7.12 R2010 software. The transient evoked responses were averaged within a 40 ms window (20 ms before and after the pulse onset). The transient latency response was determined as the time between the auditory evoked peak and the sound onset added by 2 ms (delay caused by the speaker and behavior box distance, 70 cm; Figure 3B). The response amplitude was calculated by the evoked peak value subtracted by the signal mean value from the 20 ms period before the sound onset. The signal average was used to normalize the basal value and avoid any oscillatory bias at the amplitude measurement.

The ASSR was analyzed within a 10 s window, automatically chosen from the 30 s period sound stimulus by a MATLAB algorithm. The electing method selected the window with the lowest variance value to avoid recorded movement’s artifacts. The LFP power and coherence were evaluated according to the modulation frequencies (30, 90, 150 and 210 Hz). The LFP power spectrum density (PSD—mV2/Hz) and coherence (neural activity and reference sine wave) were calculated by using MATLAB standard algorithms *pwelch* and *mscohere*, respectively (1-s window, no overlapping, chosen frequency ± 1.0 Hz). The silent period before the transient pulses was also evaluated within a 10 s window, similarly chosen by the MATLAB no-supervised algorithm. The LFP power was calculated by the whole spectrum (total energy) and separated by frequency bands (theta 4–12 Hz; beta 12–30 Hz; gamma 30–80 Hz).

### Histology

All animals from protocols “IC acoustic integration” and “IC sound processing” underwent postmortem verification of electrode placement. The brain removing and fixation follow the methodology previously published by Medeiros et al. (2014) and de Castro Medeiros et al. (2018). The brain slices (50 μm sagittal) were obtained using cryostat (Cryostat 300 e ANCAP Limited) and stained with neutral red (2%). Only data from animals with the correct IC electrode implantation were included in the subsequent statistical analysis.

### Statistical Analysis

Results were expressed as the mean ± standard error of the mean (SEM) and considered significant if *p* < 0.05. All data were analyzed using GraphPad-Prism 7^®^ (GraphPad Software). One-sample *t*-test, Unpaired *t*-test, one- and two-way analysis of variance (ANOVA; sessions as the repeated factor) followed by Bonferroni’s multiple comparisons were used according to the experimental design. The symbols * and ^#^ (Figures 1, 2) and different capital letters (Figure 3) indicate pairwise statistical significance.

## Results

### WAR Animal Displayed No Motor Deficits or Hippocampus-Dependent Memory Impairment

The rotarod task demonstrated clear training/test learning effect, but no significant difference between strains on latency for the first fall or the number of falls (first fall: Interaction Time × Groups: *F*_(1,15)_ = 2.6, *p* = 0.1; Time: *F*_(1,15)_ = 47.9, *p* < 0.0001; Groups: *F*_(1,15)_ = 1.1, *p* = 0.3; two-way ANOVA—Bonferroni’s *post hoc* test *p* > 0.05—Figure 1E; number of falls: Interaction Time × Groups: *F*_(1,15)_ = 0.9, *p* = 0.3; Time: *F*_(1,15)_ = 162, *p* < 0.0001; Groups: *F*_(1,15)_ = 0.01, *p* = 0.9; two-way ANOVA—Bonferroni’s *post hoc* test *p* > 0.05, Figure 1F). The open field also presented no statistical distinction between Wistar and WAR by the distance moved (*t*_(15)_ = 0.5, *p* = 0.5, Figure 1G) and the time moving (*t*_(15)_ = 2, *p* = 0.06, Figure 1H).

The novel object recognition (NOR) is a paradigm that evaluates hippocampal related memories with no aversive aspects (Figure 1C). The NOR’s test session demonstrated no impairment to new object recognition 24 h after training for Wistar and WAR (WAR: *t*_(7)_ = 10, *p* < 0.0001; Wistar: *t*_(9)_ = 4.8, *p* < 0.001—One-Sample *t*-Test—Figure 1D). The contextual fear conditioning test evaluated hippocampus-dependent memory regarding aversive features (Figure 1A). Both strains demonstrated a significant increase at freezing behavior in the test session compared with preconditioning, with no statistical difference between strains (Interaction Time × Groups: *F*_(1,18)_ = 0.4, *p* = 0.5; Time: *F*_(1,18)_ = 148, *p* < 0.0001; Groups: *F*_(1,18)_ = 0.2, *p* = 0.6; two-way ANOVA—Bonferroni’s *post hoc* test *p* > 0.05—Figure 1B).

### WAR Animals Exhibited No Behavior or IC Response Alteration During Classical Auditory Conditioning Phases

Wistar and WAR animals underwent to AFC by using a modulated pure tone (53.7 Hz) as CS. The paired protocol demonstrated no statistical difference for WAR animals between the preconditioning and test phases. Nevertheless, Wistar rats displayed a significant increase at freezing behavior during the test phase compared with the preconditioning and also with WAR group (test phase; Interaction Session × Groups: *F*_(1,11)_ = 7.2, *p* = 0.02; Session: *F*_(1,11)_ = 14.4, *p* = 0.02; Groups: *F*_(1,11)_ = 2.2, *p* = 0.1; two-way ANOVA—Bonferroni’s *post hoc* test *p* < 0.01 for Wistar preconditioning/test and *p* < 0.05 for Wistar and WAR test-phase—Figure 2A). The unpaired protocol exhibited no behavioral difference between sessions for Wistar animals and a higher freezing behavior at the preconditioning phase compared with the test for the WAR group (Interaction Session × Groups: *F*_(1,9)_ = 3.4, *p* = 0.1; Session: *F*_(1,9)_ = 3.8, *p* = 0.08; Groups: *F*_(1,9)_ = 1.7, *p* = 0.2; two-way ANOVA—Bonferroni’s *post hoc* test—Figure 2C). The paired/unpaired test session comparison (data not shown) also demonstrated statistical significance for Wistar (*t*_(9)_ = 4.6, *p* = 0.001) but not for WAR animals (*t*_(11)_ = 1.6, *p* = 0.1).

IC evoked responses were evaluated by LFP normalized power and coherence at 53.7 Hz. Wistar animals from the paired protocol presented a significant increase at coherence value between preconditioning and test, but no difference at LFP normalized power (Coherence Paired: Interaction Session × Groups: *F*_(1,12)_ = 4.7, *p* = 0.05; Session: *F*_(1,12)_ = 14.4, *p* = 0.002; Groups: *F*_(1,12)_ = 0.05, *p* = 0.8—Figure 2E; Coherence Unpaired: Interaction Session × Groups: *F*_(1,9)_ = 0.02, *p* = 0.8; Session: *F*_(1,9)_ = 0.2, *p* = 0.6; Groups: *F*_(1,9)_ = 6.6, *p* = 0.03—Figure 2G—two-way ANOVA—Bonferroni’s *post hoc* test *p* < 0.01 for Wistar Paired). Wistars from the unpaired paradigm and WARs (paired and unpaired) displayed no distinction between preconditioning and test sessions at LFP analyzed features (Figures 2F,G). Nevertheless, WARs demonstrated statistical higher IC normalized power compared with Wistars at both sessions of paired and unpaired protocol (Paired: Interaction Session × Groups: *F*_(1,11)_ = 0.6, *p* = 0.4; Session: *F*_(1,11)_ = 0.02, *p* = 0.8; Groups: *F*_(1,11)_ = 7.9, *p* = 0.02—Figure 2D; Unpaired: Interaction Session × Groups: *F*_(1,9)_ = 0.001, *p* = 0.9; Session: *F*_(1,9)_ = 1.2, *p* = 0.2; Groups: *F*_(1,9)_ = 13.5, *p* = 0.005—Figure 2F—two-way ANOVA).

### WAR Presents a Distinct Response to Transient Stimuli

Tone-pulse responses were evaluated by the peak latency and amplitude, as demonstrated by a representative signal from Wistar Saline (black line) and WAR Saline (red line) in Figure 3B. WAR-Saline and WAR-DZP displayed a significantly lower latency and higher amplitude response to the transient stimulus compared with Wistar groups (Latency: *F*_(4,40)_ = 12.8, *p* < 0.001; Amplitude: *F*_(4,40)_ = 18.8, *p* < 0.001; one-way ANOVA—Bonferroni’s multiple comparison test). Therefore, only the strains, but not the pharmacological manipulation, presented significant statistical modulation on the transitory evoked response. Nevertheless, it is interesting to highlight the diazepam effect in reducing the peak latency coefficient of variation at WAR DZP (3.5%), based at the WAR Saline group (6.6%), which is indicative of decreasing response jitter by the drug.

### The Animals’ Strain and the Neural Excitability Level Influence the IC Steady-State Evoked Response

Animals were acoustically stimulated by four sound modulatory frequencies (30, 90, 150 and 210 Hz), as demonstrated by the representative spectrogram in Figure 3E. The LFP power showed statistical significance only at the 150 Hz: lower values for Wistar-PTZ and WAR-Saline compared with Wistar-Saline and WAR-DZP (Interaction Frequency × Groups: *F*_(12,173)_ = 1.3, *p* = 0.2; Frequency: *F*_(3,173)_ = 5.1, *p* = 0.002; Groups: *F*_(4,173)_ = 4.4, *p* = 0.001; two-way ANOVA—Bonferroni’s *post hoc* test *p* < 0.05 for 150 Hz—Figure 3F). Nevertheless, the coherence analysis (Figure 3G) exhibited a clear group dissociation pattern from 30 Hz to 210 Hz stimulation. Although at 30 Hz all groups presented similar results, at 90 Hz the WAR-Saline animals displayed lower coherence value, differing from WAR-DZP and Wistar-DZP. The latter also showed a significantly higher value compared with Wistar-Saline, Wistar-PTZ, and WAR-Saline. At 150 Hz, the Wistar-Saline group presented an intermediate coherence value, significantly different from Wistar-DZP and WAR-DZP (upper values) and Wistar-PTZ and WAR-Saline (lower values). Under 210 Hz modulation frequency, Wistar-DZP and WAR-DZP displayed statistical higher values compared with all other groups (Wistar-Saline similar to WAR-Saline and Wistar-PTZ; Interaction Frequency × Groups: *F*_(12,173)_ = 3.4, *p* = 0.001; Frequency: *F*_(3,173)_ = 0.6, *p* = 0.6; Groups: *F*_(4,173)_ = 23.3, *p* < 0.001; two-way ANOVA—Bonferroni’s *post hoc* test *p* < 0.05—Figure 3G).

### PTZ Induces Higher Oscillatory Activity at Basal Conditions

During the silent period, the Wistar-PTZ group presented a significantly higher LFP power compared with all other groups, particularly at low-frequency bands (total power: *F*_(4,45)_ = 12.9—Figure 3H; Theta: *F*_(4,45)_ = 14.4—Figure 3I; Beta: *F*_(4,45)_ = 8.6—Figure 3J; one-way ANOVA—Tukey’s *post hoc* test *p* < 0.05). Interestingly, Wistar-DZP and WAR-DZP displayed a statistical increase of gamma power compared with Wistar-PTZ and WAR-Saline (*F*_(4,45)_ = 7.1; one-way ANOVA—Bonferroni’s *post hoc* test *p* < 0.05—Figure 3L).

## Discussion

The current work explores the seizure-free prone neural circuitry of a genetically inbred strain of rats for epilepsy WARs under seizure free conditions, and the effects it has on auditory processing and behavioral memory tasks. The WAR animals’ data demonstrated a corruption of the IC neural synchronization and impairment of classical fear conditioning, although no disturbance at the motor or hippocampus-related memory functions. The rationale for the current work was inspired by previous data that showed contradictory results regarding WARs on aversive memory tasks: enhanced performance in step-down protocols while carrying out poorly in a two-way active avoidance test (Castro et al., 2017). Although the result from the latter could be explained by the dysfunction of specific substrates processing memory, the enhanced performance in other memory tasks, known to involve some of the same neural substrates (McGaugh, 2004; Moscarello and LeDoux, 2013), makes a compelling argument against the localized dysfunction hypothesis. Another explanation, within System’s View of Epilepsy, would be that hyper-coupling and enhanced modulation by top-down hyperexcitable circuits would compromise the sensory information processing ability to isolate specific relevant cues. Therefore, non-specific transient modulation of the functional connectome would impair the formation of specific neuronal network patterns, and their facilitated propagation throughout the sensory-motor integration pathway, based on previous experience. Although it is a difficult hypothesis to test experimentally, the present work was able to extract parameters from electrophysiological and behavioral data that corroborate our claims.

Initially, Figures 1A–D depicts hippocampal-related memory tests (Broadbent et al., 2004) results for contextual conditioning and object recognition tasks, suggesting no performance difference between WARs and Wistar. Furthermore, positive controls show no indication of motor shortcomings that could influence memory task protocols (Figures 1E–H), previous corroborating data using step-down active avoidance test (Castro et al., 2017). However, WARs performed poorly on the AFC protocol (Figures 2A–C), showing similar freezing responses at paired preconditioning and test sessions (no alteration for Wistar unpaired group—Figure 2C). The ASSR was used to evaluate network integration by entraining a distinct spectral signature on circuits processing auditory information. The WAR’s elevated power (ASSR frequency) in the preconditioning and test phases (paired and unpaired protocols), suggests an increased recruitment of neural networks processing the sound stimuli (Figures 2D,F); which could also be explained by higher synchronicity in neuronal population discharge (Kudela et al., 2003; Medeiros and Moraes, 2014) and confirms previous findings from our laboratory (Pinto et al., 2017). Nevertheless, the coherence data does not show significantly enhanced (Figures 2E,G) when comparing Wistar and WARs. In addition, the Wistar paired protocol (Figure 2E) during the Test phase had a significant increase in coherence, indicating that previous experience had a modulatory effect on downstream IC processing, possibly by facilitating bottom-up ascending neuronal coupling of that specific learned cue. These results also confirm published data that have used ASSR to evaluate changes in the downstream processing of sensory stimuli in classical fear conditioning protocols (Lockmann et al., 2017). It is important to highlight that more rostral areas receiving projections from the auditory pathway, such as the amygdaloid complex, have shown plastic changes associated with memory acquisition in fear conditioning tasks (Maren, 2001). Thus, the phase-locking of IC neuronal activity with auditory stimulation may be an effect of top-down modulation from more rostral circuits, that have been plastically changed by the experience, facilitating the propagation of specific relevant cues throughout the primary auditory pathway. In fact, if such a top-down modulation was unable to generate the specific activity patterns that would yield the facilitation of upstream transference of relevant cues, a decreased stability in the ASSR response would be expected within the IC. That being the case, only increasingly non-specific facilitation would be likely to produce proper sensory-motor integration. These hypotheses were further investigated in the experiments summarized in Figure 3.

First, to evaluate the IC bottom-up response, with minimal interference from top-down circuitry, the transient evoked response was recorded from both strains under different pharmacological treatments. The Wistar IC evoked response peak latency (5.5 ± 0.1 ms) is within what has been published elsewhere (Moraes et al., 2000; Figure 3C). The shorter latency and increased amplitude of WARs evoked response (Figures 3C,D) suggest a hyperexcitable and facilitated primary auditory pathway that may play an important role in the reflex seizures triggered by high-intensity sound stimulation (Garcia-Cairasco, 2002; Ribak, 2017). The fact that neither DZP or PTZ (for Wistars) significantly altered the transient evoked response amplitude or latency is suggestive that these drugs may have a pronounced effect on more rostral circuitry. In fact, brain mapping studies of DZP (Zezula et al., 1988; Griessner et al., 2018) and PTZ (André et al., 1998; Mesquita et al., 2011) support the interpretation we have given to these results, highlighting the effect on the cortex and limbic system structures. Nevertheless, the jitter of peak latency for WARs has a tendency of diminishing after DZP treatment (as described in the “Results” section). Altogether, this could suggest that WAR’s top-down tonic modulation of the IC could be unspecifically enhanced due to genetic selection for seizures, a hypothesis that may also be raised using kindling experimental approaches (Garcia-Cairasco et al., 1996; Dutra Moraes et al., 2000).

The rationale behind using four different modulating frequencies for the ASSR stimuli was as follows: (a) 30 Hz stimuli falls within the higher EEG energy bandwidth, theoretically more susceptible to endogenous oscillator interference; (b) the 90 Hz is in gamma range higher end, thus expected to have a better signal to interference ratio regarding the spectral signature (Buzsáki, 2006); (c) 150 Hz would have the best spectral signal/interference ratio due to low EEG baseline energy but still not too fast to compromise network entrainment; and (d) 210 Hz driving circuitry would be restrained by the longer synaptic delays, generating a quasi-sustained modulation of the network and reducing the distinguish of peaks and valleys. Our results show that at 30 Hz modulation there were no statistical differences within groups, most likely due to increased variability and strong interference from the bulk of baseline EEG oscillations. Nevertheless, DZP (Wistar and WAR) seems to have elevated power at the modulating frequency (Figure 3F), possibly due to its effect on gabaergic feedback mechanisms (van Lier et al., 2004; Baumgarten et al., 2016). Conversely, PTZ for Wistars seemed to have had the same effect (Figure 3F); however, if one considers its effect on Total Energy as well (Figures 3H–K), it is likely due to non-specific increased excitability within the 30 Hz spectral range. The 90 Hz modulation frequency of WARs has a clear increase in energy, particularly when normalized by baseline EEG (graph not shown—90 Hz normalized energy: *t*_(17)_ = 2.2, *p* = 0.03—Unpaired *t*-test, Wistar-Saline, and WAR-Saline) corroborating results from the transient response (Figure 3C) and previous work from our group (Pinto et al., 2017). In addition, DZP promoted a significant increase in coherence (Figure 3D) for both strains, suggesting that gabaergic system enhancement facilitates the network entrainment by external stimuli, possibly by compromising IC top-down modulation. In fact, DZP action in the Amygdaloid Complex, which receives IC projections through the Medial Geniculate Body (Weinberger, 2011), is known to compromise auditory memory formation, consolidation, and retrieval (Makkar et al., 2010). The top-down modulation of AMY-IC projections may play a critical role in a pattern specific facilitation of auditory cues throughout the primary sensory pathway (Maisonnette et al., 1996; Macedo et al., 2006), a key element in memory task performance. The 150 and 210 Hz stimuli show that WAR and Wistar-PTZ present similar ASSR profiles for energy and coherence (Figures 3F,G), suggesting that WAR’s basal tonic IC modulation from top-down structures could be unspecifically potentiated. Furthermore, under DZP, coherence for both strains rise to almost perfect entrainment (Figure 3G). When comparing Wistar ASSR power between modulation frequencies of 150 and 210 Hz, the effect of quasi-sustained modulation of the network becomes evident. In fact, 150 Hz stimulated Wistar-PTZ show similar power (Sugaya et al., 1987; Medeiros et al., 2014) responses at its specific spectral signature than 210 Hz Wistar-Saline. The total power of baseline EEG showed higher values for Wistar-PTZ, as expected from its excitatory effect on neural circuitry (Sugaya et al., 1987; Medeiros et al., 2014), and, except for WAR-DZP gamma power increase (also corroborating stronger top-down modulation on local circuitry), remained fairly unchanged among the other groups.

Altogether, the results suggest that the WAR seizure prone neural system may have a non-specific tonically enhanced top-down modulation of IC circuitry, which interferes with sensory information processing and may lead to poor performance in tasks that require the identification of specific patterns elicited by an auditory cue. Additional experiments, including discrimination paradigms, are necessary to strengthen and expand the arguments for the top-down regulation of the IC and its involvement in cognitive functions. Nevertheless, if these results are proven to be a generalized property of seizure predisposed networks, the ASSR could be a useful diagnostic tool to access the imbalance of the neural system excitatory/inhibitory tonus, condition closely associated with the disturbance of networks synchronization and with the ictal hypersynchronous state. Additionally, the external driven (modulated sound) oscillatory activity might support the evaluation of epilepsy-associated cognitive comorbidities, particularly those involving the memory plastic changes.

## Data Availability Statement

The datasets generated for this study are available on request to the corresponding author.

## Ethics Statement

The animal study was reviewed and approved by Ethical Committee for the Use of Animals (CEUA)–Universidade Federal de Minas Gerais. License number 112/2014.

## Author Contributions

DM and MM planned the studies, wrote the first draft of the article and obtained funding. HP, EO, VC, FM, LG and DM conducted experiments. FM, EM, DM and MM interpreted the results. DM and MM wrote the first draft of the article. All authors reviewed, edited and approved the article.

## Conflict of Interest

The authors declare that the research was conducted in the absence of any commercial or financial relationships that could be construed as a potential conflict of interest.
